# P-758. A Comprehensive Analysis of Multi-Drug Resistant Tuberculosis: A Population-Based Time-Trend Analysis

**DOI:** 10.1093/ofid/ofae631.953

**Published:** 2025-01-29

**Authors:** Ahmed Husain, Saqr Alsakarneh, Lujain Malkawi, Mohd Alghizzawi, Christopher Brychel

**Affiliations:** University of Missouri - Kansas City, Kansas City, MO, USA., Kansas City, Missouri; University of Missouri - Kansas City, Kansas City, MO, USA., Kansas City, Missouri; University of Missouri - Kansas City, Kansas City, MO, USA., Kansas City, Missouri; University of Missouri - Kansas City, Kansas City, MO, USA., Kansas City, Missouri; University of Missouri - Kansas City, Kansas City, MO, USA., Kansas City, Missouri

## Abstract

**Background:**

Due to the significant morbidity and mortality that it carries, Multi-Drug resistant Tuberculosis (MDR-TB) remains a pressing concern for public health. The aim of this study was to conduct a time-trend analysis of age and gender-specific MDR-TB incidence rates in the United States (US) using the Global Burden Diseases (GBD) 2019 database.

**Figure:**
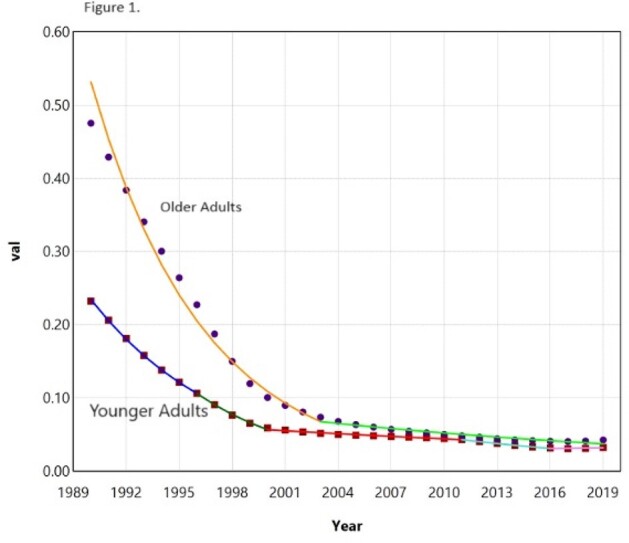

**Methods:**

Data was obtained from the GBD 2019 database, an international database that encompasses ∼100% of TB diagnosed cases in the US. TB incidence rates, age-adjusted to the standard US population, were calculated using SEER*Stat software (v.8.4.0.1, National Cancer Institute “NCI”) and were additionally stratified according to sex. Time-trends were estimated as an Annual Percentage Change (APC) and Average APC (AAPC) using Joinpoint Regression Software (v.4.9.0.1, NCI) utilizing Monte Carlo permutation analysis to generate the simplest trend. Pairwise comparison was conducted between gender-specific trends using the tests of parallelism and coincidence. Age-specific trends were also assessed in two age sub-groups: younger adults 15-49 years of age and older adults 50-74 years of age. A two-sided P-value cut-off of 0.05 was utilized for statistical significance.

**Figure:**
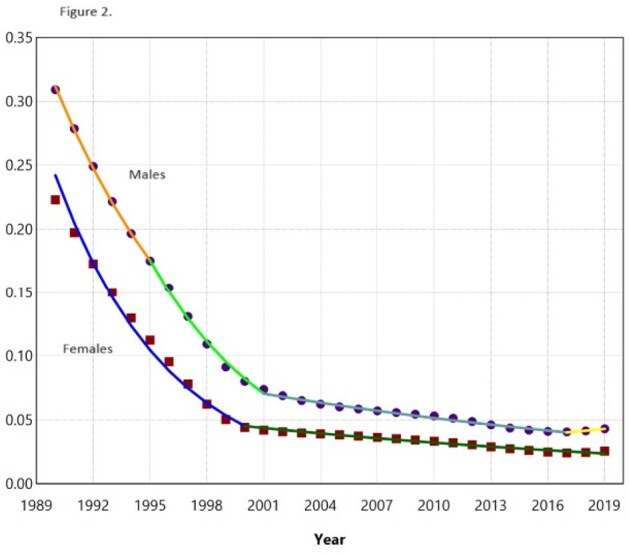

**Results:**

6,820 patients were diagnosed with MDR TB in the US between 1990-2019. Overall, MDR-TB incidence rates have decreased in all study groups, with a more substantial decrease in older adults than younger adults (AAPC= -8.7 vs -6.6; AAPC difference=1.2, P< 0.0001). Age-specific trends were neither identical (P< 0.001) nor parallel (P< 0.001) (Figure 1), suggesting that MDR-TB incidence rates are different and decreasing at a greater rate in older adults compared to younger adults. Similarly, the incidence of MDR-TB has shown a greater reduction in females than males over the review time period (AAPC= -7.7 vs -6.6; AAPC difference= 1.1, P< 0.0001) (Figure 2).

**Conclusion:**

Our nationwide study demonstrated that MDR-TB incidence trends have been decreasing in the United States over the past three decades, with a greater reduction noted in older adults and females.

**Disclosures:**

**All Authors**: No reported disclosures

